# *Rickettsia japonica* Infections in Humans, Zhejiang Province, China, 2015

**DOI:** 10.3201/eid2411.170044

**Published:** 2018-11

**Authors:** Qunying Lu, Jianping Yu, Liqun Yu, Yanjun Zhang, Yitao Chen, Meiai Lin, Xiaofei Fang

**Affiliations:** Zhejiang Province Center for Disease Control and Prevention, Hangzhou, China (Q. Lu, Y. Zhang, X. Fang);; Linan First People’s Hospital, Linan, China (J. Yu, L. Yu);; Zhejiang Chinese Medical University, Hangzhou (Y. Chen, M. Lin)

**Keywords:** spotted fever group rickettsiae, *Rickettsia japonica*, human infection, fever, rash, lymphadenopathy, China, *groES*, *groEL*, *ompA*, tickborne infection, vector-borne infections, bacteria, Zhejiang Province, Japanese spotted fever, ticks

## Abstract

We investigated 16 Japanese spotted fever cases that occurred in southeastern China during September–October 2015. Patients had fever, rash, eschar, and lymphadenopathy. We confirmed 9 diagnoses and obtained 2 isolates with high identity to *Rickettsia* *japonica* strain YH. *R. japonica* infection should be considered for febrile patients in China.

*Rickettsia japonica* is a member of the spotted fever group rickettsiae (SFGR) that causes tickborne Japanese spotted fever (JSF). First reported in Japan’s Tokushima Prefecture in 1984 (*1*,*2*), JSF has been recognized in multiple countries of Asia, including Japan, South Korea, the Philippines, and Thailand (*3**–**5*). In China, 4 species of SFGR have been reported to cause human infection: *R. heilongjiangensis*, *R. sibirica* subspecies *sibirica* BJ-90, *Candidatus* R. tarasevichiae, and *R. raoultii* (*6*). In this report, we investigated the causative agent of 16 JSF cases that occurred in southeastern China in late 2015.

## The Study

The ethics committee of the Zhejiang Province Center for Disease Control and Prevention, Hangzhou, China, approved this research. During September–October 2015, a total of 16 febrile patients were hospitalized at Linan First People’s Hospital (Linan, China). All these patients lived in the Xitianmu Mountain area of Linan in Zhejiang Province. Besides fever (38.8°C–40.3°C), clinical signs of disease in these patients included rashes on the trunk and limbs (15/16) and an eschar (11/16) (Table). In those with eschar, lymphadenopathy was found at the site of the draining lymph node (6/11). Five patients had rash and no eschar. Laboratory results revealed that all patients’ leukocyte levels were within reference ranges, but a high percentage of neutrophils (12/16 patients) and minor hepatic transaminase elevation (11/16 patients) were also observed. All 16 patients were treated with doxycycline or azithromycin and were cured, and no patient experienced severe illness.

**Table:** Clinical data of febrile patients with Japanese spotted fever diagnoses, Linan, China, 2015*

Patient no.	Age, y/sex	Tick bite	Rash	Eschar	Lymphadenopathy	Days after onset		PCR,† AP	IFA titer‡		Isolation
						AP	CP		AP	CP	
1	53/M	Not known	Yes	No	No	6	42	–	<64	4,096	–
2	62/M	Not known	Yes	Yes	No	5	58	+	<64	2,048	–
3	52/M	Yes	Yes	Yes	Yes	5	74	+	64	4,096	–
4	79/F	Not known	Yes	No	No	4	60	+	128	9,192	+
5	67/F	No	Yes	No	No	6	75	+	512	4,096	–
6	62/F	Yes	Yes	Yes	No	7	70	–	<64	1,024	–
7	53/F	Yes	No	Yes	No	7	57	–	64	1,024	–
8	63/F	Yes	Yes	Yes	Yes	10	67	–	<64	4,096	–
9	57/M	Yes	Yes	Yes	No	2	45	+	64	9,192	–
10	51/F	Yes	Yes	Yes	Yes	6	§	+	64	§	–
11	53/M	Yes	Yes	Yes	No	3	§	+	<64	§	–
12	45/F	Yes	Yes	Yes	Yes	5	70	+	<64	4,096	–
13	33/F	Yes	Yes	Yes	Yes	6	42	–	64	2,048	–
14	30/F	Not known	Yes	No	No	4	46	–	128	2,048	–
15	36/F	Not known	Yes	No	No	5	54	–	<64	2,048	–
16	67/F	Yes	Yes	Yes	Yes	0	60	+	<64	9,192	+

*AP, acute phase; CP, convalescent phase; IFA, indirect immunofluorescence assay; +, positive test result; –, negative test result. †PCR amplifying 217-bp sequence of *Rickettsia japonica groEL* gene. ‡Detection of IgG against spotted fever group rickettsiae. §Convalescent-phase serum samples not obtained.

With patient consent, we collected acute-phase (n = 16) and convalescent-phase (n = 14) whole blood specimens and sent them to Zhejiang Province Center for Disease Control and Prevention for laboratory confirmation of *Rickettsia* infection. We extracted DNA from acute-phase blood specimens by using the DNeasy Blood & Tissue Kit (QIAGEN, Hilden, Germany). We used nested PCR to amplify the partial *groEL* genes of SFGR, typhus group rickettsiae, and *Orientia tsutsugamushi* bacteria (Technical Appendix). The targeted fragments (217 bp) were present in the blood specimens from 9 patients (Table). We sequenced these fragments and analyzed them using BLAST (http://www.ncbi.nlm.nih.gov/BLAST), and all had 100% identity to *R. japonica* YH prototype strain (GenBank accession no. NC016050) (*7**,**8*). All specimens were negative for typhus group rickettsiae and *O. tsutsugamushi* rickettsia DNA by PCR.

We also inoculated 200 μL of acute-phase blood specimens onto HL60 and DH82 cells in 25-mL flasks and cultured at 37°C. Cytopathic effect was not observed with inoculated HL60 cells, but inoculated DH82 cells exfoliated completely by 4 weeks of culture. We also performed indirect immunofluorescence assays (IFAs) every 2 days to access SFGR growth (Technical Appendix). Two of the inoculated cultures exhibited bright fluorescent apple-green, rod-shaped particles (Table) after 3 weeks of culture, confirming SFGR infection for 2 patients. We then extracted DNA from the 2 SFGR-positive cultures (LA4/2015 and LA16/2015) and amplified and sequenced a 2,493-bp fragment containing the full-length sequences of SFGR *groES* and *groEL* (GenBank accession nos. KY073364–5) and a 609-bp fragment containing the partial rickettsial *ompA* gene sequence (GenBank accession nos. KY347792–3; Technical Appendix Table). These sequences were found to be 100% identical to the corresponding sequences of *R. japonica* YH.

We used IFAs with bacterial substrate antigens *R. japonica* (HL-60 cells infected with LA4/2015) and *R. rickettsii* (FOCUS Diagnostics Inc., Cyprus, CA, USA) to test patients for specific antibodies, and in all 16 patient serum samples, we detected SFGR IgG. All paired serum samples (n = 14) showed a >4-fold increase in titer against SFGR (Table). The 2 patients we did not receive convalescent-phase serum specimens from were positive for *R. japonica* by PCR.

All serum specimens were negative for *O. tsutsugamushi* IgG. Some convalescent-phase serum specimens had low-titer reactions to *R. typhi* bacterial antigen.

## Conclusions

The 4 SFGR species *R. japonica*, *R. heilongjiangensis*, *R. rhipicephali*, and *R. massiliae* have been identified in *Haemaphysalis longicornis* and *Rhipicephalus haemaphysaloides* ticks in Zhejiang Province (*9**–**11*), indicating a potential for these species to infect humans in China. In our research, we determined the etiologic agent of 16 JSF cases and isolated 2 *R. japonica* rickettsiae. The prototype strain *R. japonica* YH was isolated in Japan in 1985 (*1*). After ≈30 years, only a few *R. japonica* isolates have been isolated from patients in China: 2 from our research and 1 from Li et al. (*12*). Our findings indicate that the full-length *groES* and *groEL* genes and the partial *ompA* gene sequences were 100% identical to *R. japonica* YH, suggesting that the *R. japonica* genome has been relatively conserved. Nine patients had clinically confirmed JSF, displaying fever, rash, eschar, and lymphadenopathy; these signs and symptoms were similar to those seen in JSF patients in Japan (*13*).

Of the vectorborne rickettsial diseases in China, human scrub typhus and murine typhus frequently occur in Zhejiang Province, and spotted fever group rickettsiosis probably occurs but has gone relatively unnoticed. Because the clinical symptoms of spotted fever and scrub typhus are similar, some SFGR infections have likely been diagnosed as scrub typhus. We found that the blood specimens of 7 febrile patients were negative for the targeted PCR fragments but showed a >4-fold increase in antibody titer to SFGR. Although these results suggest SFGR infection, we cannot conclude these 7 patients were infected with *R. japonica*.

In summary, *R. japonica* infections occur in Zhejiang Province, China. These infections are likely more broadly distributed throughout the mainland areas than had been previously realized. Improvements in JSF clinical diagnosis and human epidemiologic surveillance are urgently needed in China.

Technical Appendix Primer information and description of methods used for febrile patients with *Rickettsia japonica* infections, China, 2015.
