# Supplementary material for: Rickettsia japonica Infections in Humans, Zhejiang Province, China, 2015
Source: Emerg Infect Dis. 2018 Nov;24(11):2077–9. doi: 10.3201/eid2411.170044 (PMC6200003; doi:10.3201/eid2411.170044)
Supplement: Technical Appendix — Primer information and description of methods used for febrile patients with Rickettsia japonica infections, China, 2015. [file 17-0044-Techapp-s1.pdf]

Article DOI: https://doi.org/10.3201/eid2411.170044

Rickettsia japonica Infections in Humans, Zhejiang Province, China, 2015

Technical Appendix

Materials

Molecular Detection of Rickettsial Infection

For laboratory confirmation of *Rickettsia* infection, patient whole blood specimens were collected during the acute phase and sent to Zhejiang Province Center for Disease Control and Prevention, Hangzhou, China. DNA extraction and PCR were performed in a standard PCR laboratory and each step included negative controls. DNA was extracted from acute phase blood specimens and cell culture by using the DNeasy Blood & Tissue Kit (QIAGEN, Hilden, Germany) according to the manufacturer's instructions.

First, nested PCR of the *groEL* gene (217 bp) of spotted fever group rickettsiae (SFGR), typhus group rickettsiae, and *Orientia tsutsugamushi* bacteria were performed as described previously (*1*,*2*). PCR targeting the SFGR *ompA* gene was performed to identify the bacteria grown in cell culture as described previously (*3*). Another PCR was performed with primers specific to the nucleotide sequences of the full-length SFGR *groES* and *groEL* genes (Technical Appendix Table). The sequences were aligned and trimmed with MEGA 5.0 (https://www.megasoftware.net/) (*4*).

Indirect Immunofluorescence Assay (IFA) Detection for SFGR in Inoculated Cells

In total, 200 μL of blood samples (collected with K2 EDTA tubes) from every patient were inoculated onto HL60 and DH82 cells and cultured at 37°C. Slides of inoculated and

noninoculated (negative control) cells were prepared and fixed in cold acetone for 7 minutes; IFA was used to determine whether the inoculated cells were infected (*5*). SFGR positivity of the 2 convalescent patient serum samples was confirmed by *Rickettsia* IFA IgG (FOCUS Diagnostics Inc., Cypress, CA, USA), and then, these positive serum samples were used as the primary antibody for SFGR-specific IFAs, with goat anti-human IgG (Sigma-Aldrich, St. Louis, MO, USA) serving as the secondary antibody.

References

1. Park HS, Lee JH, Jeong EJ, Kim JE, Hong SJ, Park TK, et al. Rapid and simple identification of *Orientia tsutsugamushi* from other group rickettsiae by duplex PCR assay using *groEL* gene. Microbiol Immunol. 2005;49:545–9. PubMed http://dx.doi.org/10.1111/j.1348-0421.2005.tb03760.x

2. Fu XP, Zhang JS, Shen XJ, Luan MC, Li ML, Zhang LJ. Surveillance on *Rickettsia* in epidemic areas of scrub typhus in Xinyang areas of Henan [in Chinese]. Zhonghua Liu Xing Bing Xue Za Zhi. 2007;28:547–9. PubMed

3. Zhang L, Jin J, Fu X, Raoult D, Fournier P-E. Genetic differentiation of Chinese isolates of *Rickettsia sibirica* by partial *ompA* gene sequencing and multispacer typing. J Clin Microbiol. 2006;44:2465–7. PubMed http://dx.doi.org/10.1128/JCM.02272-05

4. Tamura K, Peterson D, Peterson N, Stecher G, Nei M, Kumar S. MEGA5: molecular evolutionary genetics analysis using maximum likelihood, evolutionary distance, and maximum parsimony methods. Mol Biol Evol. 2011;28:2731–9. PubMed http://dx.doi.org/10.1093/molbev/msr121

5. Uchida T, Uchiyama T, Kumano K, Walker DH. *Rickettsia japonica* sp. nov., the etiological agent of spotted fever group rickettsiosis in Japan. Int J Syst Bacteriol. 1992;42:303–5. PubMed http://dx.doi.org/10.1099/00207713-42-2-303

Technical Appendix Table. Information on primers used for PCR and sequencing in study of febrile patients with Japanese spotted fever, Linan, China, 2015*

Organism	Target gene	Primer	Sequence	Reference
Rickettsiaceae	*groEL*	GRO1	5′-AAGAAGGMGTGATAAC-3′	(*1*)
		GRO2	5′-ACTTCMGTAGCACC-3′	
Rickettsia	*groEL*	SF1	5′-GATAGAAGAAAAGCAATGATG-3′	(*2*)
		SR2	5′-CAGCTATTTGAGATTTAATTTG-3′	
Orientia tsutsugamushi	*groEL*	TF1	5′-ATATATCACAGTACTTTGCAAC-3′	
		TR2	5′-GTTCCTAACTTAGATGTATCAT-3′	
Spotted fever group rickettsiae	*groES* and *groEL*	F1	5′-CTCCGAATAGTTTAGGTAATTGGC-3′	This research
		R1	5′-TTTCGGTRCCTGCCCATTTAC-3′	
		F2	5′-GGTGAAGAGAAAACTAAAGGTGGA-3′	
		R2	5′-CTGCTAAATCCATACCACGCTT-3′	
		F3	5′-TGCAGAGGTAGCHGGTGAYGG-3′	
		R3	5′-TAACATTTTCAAGCTTCATACCTA-3′	
		F4	5′-CGCTTGTAGTCAATAGATTACGTGG-3′	
		R4	5′-TACTAGATCTAYTRCATAACCTATCTT-3′	
	ompA	Rr190.70p	5′-ATGGCGAATATTTCTCCAAAA-3′	(*3*)
		Rr190–701R	5′-GTTCCGTTAATGGCAGCATCT-3′	

*Primers GRO1 and GRO2 were used in the first round for nested PCR. SF1 and SR2, specific to *Rickettsia*, were used in the second round of PCR. TF1 and TR2 are specific to *Orientia tsutsugamushi.* Primers F1, R1, F2, R2, F3, R3, F4, and R4 refer to the sequence of *R. japonica* YH (GenBank accession no. NC_016050).
